# Brain Cholesterol Metabolism and Its Defects: Linkage to Neurodegenerative Diseases and Synaptic Dysfunction

**Published:** 2016

**Authors:** A. M. Petrov, M. R. Kasimov, A. L. Zefirov

**Affiliations:** Kazan Medical University, Department of Normal Physiology, Butlerova str. 49, Kazan, Russia, 420012

**Keywords:** lipid rafts, neurodegenerative disease, oxysterols, synaptic transmission, cholesterol

## Abstract

Cholesterol is an important constituent of cell membranes and plays a crucial role in the compartmentalization of the plasma membrane and signaling. Brain cholesterol accounts for a large proportion of the body’s total cholesterol, existing in two pools: the plasma membranes of neurons and glial cells and the myelin membranes . Cholesterol has been recently shown to be important for synaptic transmission, and a link between cholesterol metabolism defects and neurodegenerative disorders is now recognized. Many neurodegenerative diseases are characterized by impaired cholesterol turnover in the brain. However, at which stage the cholesterol biosynthetic pathway is perturbed and how this contributes to pathogenesis remains unknown. Cognitive deficits and neurodegeneration may be associated with impaired synaptic transduction. Defects in cholesterol biosynthesis can trigger dysfunction of synaptic transmission. In this review, an overview of cholesterol turnover under physiological and pathological conditions is presented (Huntington’s, Niemann-Pick type C diseases, Smith-Lemli-Opitz syndrome). We will discuss possible mechanisms by which cholesterol content in the plasma membrane influences synaptic processes. Changes in cholesterol metabolism in Alzheimer’s disease, Parkinson’s disease, and autistic disorders are beyond the scope of this review and will be summarized in our next paper.

## CHOLESTEROL RECYCLING IN THE BRAIN

**The pools of cholesterol in the brain**

Cholesterol is a major lipid component of brain cell membranes, accounting for 23–25% of the body’s total cholesterol content. The brain has cholesterol content at 15–30 mg/g tissue, whereas the average in other tissues is at 2–3 mg/g tissue [1]. In the central nervous system (CNS), cholesterol has a number of essential functions. Cholesterol-enriched myelin sheaths serve as an insulation layer increasing nerve conduction velocity. Cholesterol is abundantly present in the synaptic membranes to aid in nerve signal transmission. Cholesterol deficiency has been shown to inhibit dendrite growth [2, 3].

A total of over 30 enzymes catalyze the synthesis of cholesterol in mammals. Outside the CNS, cholesterol can be synthesized *de novo *(about 50–60%), or it can be obtained from the diet (lipoprotein-bound). However, the blood brain barrier (BBB) prevents the uptake of lipoprotein- bound cholesterol from the circulation. Most brain cholesterol (over 95%) comes from *in situ *synthesis mainly in glial cells [1]. Increased permeability of BBB to sterol molecules is related to BBB impairment [4]. Partial disruption of BBB may be a result of aging. In addition, the function of the BBB can be significantly affected in neurodegenerative disorders, which can ultimately lead to pathological conditions [5, 6]. Using pericyte-deficient mice, which are critical to BBB functioning, an age-related progressive neurodegenerative disease was observed [4, 6].

Brain cholesterol is found in two major stores. The smaller one is subject to relatively fast turnover rates (half-life of 5–10 months, 8 mg/g) and made up by the plasma membranes of neurons (10%) and glial cells (20%). The larger pool of CNS cholesterol (70%) is in myelin (40 mg/g) with very slow turnover (half-life of approximately 5 years) [7]. The rate of cholesterol synthesis is highest during the period of active myelination by oligodendrocytes (the first few weeks/months after birth). Oligodendrocytes use ketone bodies as precursors for lipid synthesis (ketone-metabolizing enzymes), whose plasma levels are 10-fold increased during myelin sheath formation. Cholesterol-deficient oligodendrocytes show dependence on the local supply of extracellular cholesterol, dramatically reducing CNS myelination [8]. Following myelination the production of cholesterol drops by 90%, and in the mature brain it only occurs in astrocytes and neurons, albeit at a 5-fold slower rate than in astrocytes [1]. Neuronal cholesterol biosynthesis plays a crucial role in the survival and differentiation of axons and dendrites, and formation of non-efficient synapses. The *de novo *synthesis of cholesterol in neurons can be upregulated by the brain-derived neurotrophic factor (BDNF) [9]. This period of extensive synapse formation (particularly, presynaptic terminals away from the some) requires astrocyte-derived cholesterol. Cultured neurons elicit a 10-fold increase in excitatory synaptic transmission and generate 5–7 fold greater synapses in the presence of astrocytes, suggesting a role for astrocyte-derived cholesterol in neuronal function. Overall, the synthesis of cholesterol by neurons is essential to the developing brain, whereas in the adult brain neurons rely on external sources of cholesterol [1, 7].

**Regulation of cholesterol synthesis**

**Fig. 1:**
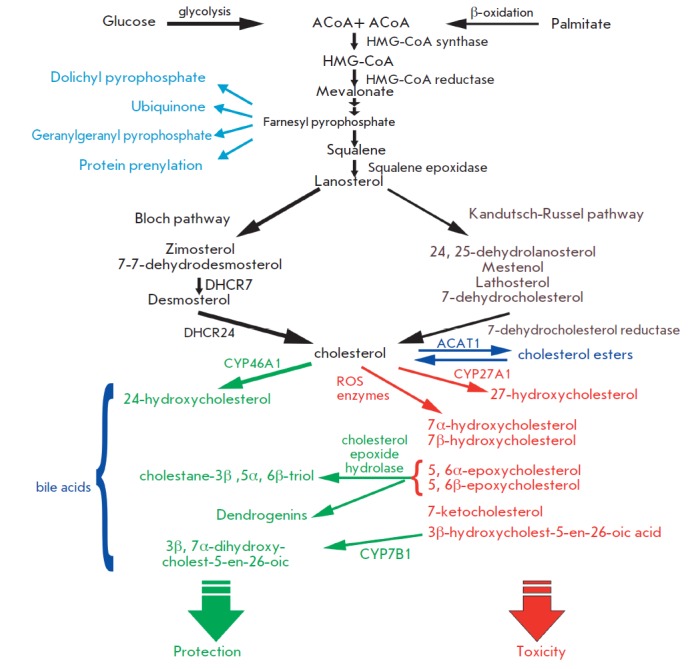
Cholesterol synthesis and oxysterol formation. Cholesterol is produced from acetyl-coenzyme A in a multistage enzymatic process. There are two pathways for cholesterol synthesis; the Bloch and Kandutsch-Russel pathways. *De novo *synthesized cholesterol can accumulate as cholesterol esters or be modified by enzymic or non-enzymic oxidation into oxysterols. A wide array of oxysterols have been described, each of which may have a specific effect on cellular functions. See text for a detailed explanation.

**Fig. 2:**
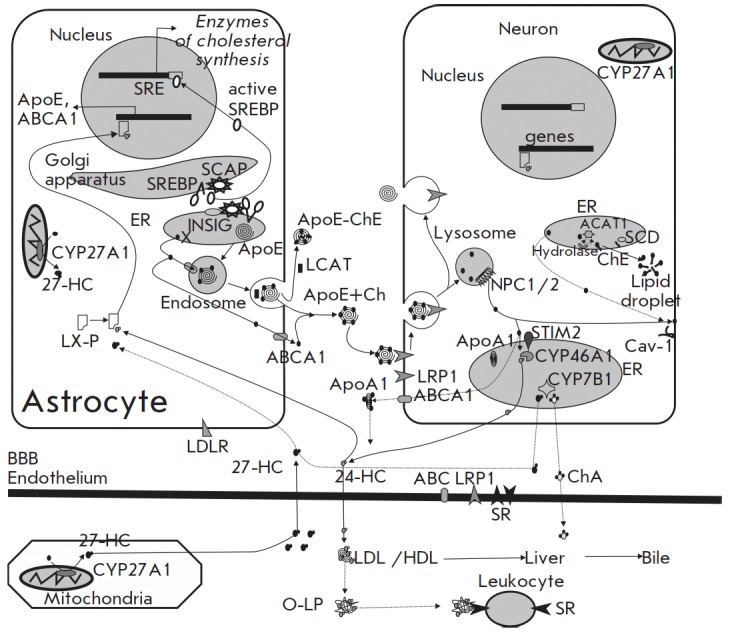
Brain cholesterol metabolism: neuron–glial interplay. The major input of cholesterol into the brain comes from* in situ *synthesis in the endoplasmic reticulum (ER) of astrocytes. The proteins INSIG, SREBP, and SCAP regulate the cholesterol biosynthetic machinery. These proteins are tightly associated and retained in the ER at high levels of sterols. When sterol levels drop below a threshold, the complex dissociates, allowing SREBP and SCAP to translocate to the Golgi apparatus. Within this organelle, SCAP cleaves SREBP, releasing the active transcription factor, which then migrates to the nucleus to activate the genes involved in cholesterol synthesis and trafficking. Lipoprotein particles, including apolipoproteins E (ApoE), assembled in the ER are targeted to endosomes for secretion into the extracellular space. Newly synthesized cholesterol is transported from the ER to endosomes or extracellular space by non-vesicular mechanisms via ATP-binding cassette transporters (ABCA1). Cholesterol-rich ApoE-particles interact with the neuronal receptors (LRP1), undergo internalization by receptor-mediated endocytosis, and are routed to late endosomes/lysosomes. Once there, NPC1 /2 proteins promote trafficking of cholesterol to the plasma membrane or the ER. The supply of the plasma membrane with cholesterol requires caveolin-1 (Cav-1). Membrane cholesterol could be processed by CYP46A1 to 24-hydroxycholesterol (24-HC), which passes through the blood-brain barrier (BBB) and binds to light or high density lipoproteins (LDL or HDL). Increased plasma levels of 24-HC can oxidize the plasma lipoproteins (O-LP) that are then accumulated in leukocytes via scavenger receptor (SR) mediated endocytosis. Binding of 24HC to the cytoplasmic LX-receptors of astrocytes (or neurons) triggers expression of the genes (ApoE and ABCA1) involved in cholesterol trafficking from astrocytes to neurons. A certain amount of cholesterol can exit the brain through the BBB in the form of ApoA1-partciles. Elevated cholesterol content in the ER upregulates an ACAT1-dependent generation of cholesterol esters, which build up in the cytoplasm as lipid drops. SCD (stearoyl-CoA desaturase) supplies the monounsaturated fatty acids required for cholesterol esterification. Accumulation of cholesterol esters (as ApoE-particles, ApoE-ChE) in the extracellular space is associated with LCAT (lecithin–cholesterol acyltransferase) activity secreted by astrocytes. Mitochondria of many cells (in particular, macrophages) have the enzyme CYP27A1 that catalyzes the conversion of cholesterol to 27-hydroxycholesterol (27-HC) that could transverse the BBB and less effectively (as compared to 24-HC) activate LX-receptors. The neuronal enzyme CYP7B1 can convert 27-HC to 7α-hydroxy-3-oxo-4-cholestenoic acid (ChA) cleared from the brain into the circulation. Although the BBB is not permeable to plasma cholesterol, BBB endothelial cells make possible cholesterol flux across the BBB via ABC transporters and LRP1 and SR.

*De novo *cholesterol synthesis begins with the transformation of acetyl-CoA into 3-hydroxyl-3-methylglutaryl- coenzyme A (HMG-CoA) via a reaction catalyzed by HMG-CoA -synthetase and then by HMG-CoA reductase into mevalonate. The HMG-CoA reductase-catalyzed formation of mevalonate is an irreversible and rate-limiting step in the cholesterol biosynthesis, targeted by statin drugs. There are two cholesterologenic pathways in the brain (*Fig. 1*). Neurons mainly contain sterols synthesized via the Kandutsch-Russel cholesterol synthetic pathway (7-dehydrocholesterol, lanosterol), and astrocytes contain precursors of the Bloch pathway (desmosterol) [10]. The machinery of cholesterol synthesis resides in the endoplasmic reticulum (ER). The cholesterol content in the ER shows greater variations than in plasma membranes. Indeed, the cholesterol environment in the ER influences the total cholesterol levels in the cell. One of the key players in cholesterol regulation is SREBP-2 (sterol-regulatory element-binding protein), an inactive transcription factor anchored to the ER membrane and capable of binding to SCAP (SREBP cleavage-activating protein), which functions as a detector of cholesterol due to a sterol-sensing domain. During high cholesterol concentrations, the SREBP-2/SCAP complex is retained in the membranes of the ER by the retention proteins INSIG- 1 and -2 (insulin-induced protein 1 and 2). In sterol- depleted cells, the interaction between the INSIG retention complex and SREBP-2/SCAP is lost, allowing SCAP to escort SREBP-2 to the Golgi compartment. Within this organelle, SCAP releases the N-terminal domain of SREBP-2, which translocates to the nucleus to bind sterol regulatory elements (SRE) in the promoter regions of over 30 target genes encoding enzymes of cholesterol biosynthesis (*Fig. 2*) [1, 10-12].

SCAP knockout mice show a 30–40% reduction in brain cholesterol synthesis, leading to defects in synaptic transmission [13]. Disruption of the SCAP gene in astrocytes results in microcephaly, motor deficits and behavioral dysfunctions, which could be partially rescued by the uptake of dietary lipids [14]. Schwann cell SCAP mutant mice exhibit congenital hypomyelination and neuropathy-related behavior, tremor, and abnormal gait [15]. The inhibition of cholesterol synthesis reduces the expression of cholesterol-binding proteins, such as myelin proteins [8].

Newly synthesized cholesterol leaves the ER by vesicular and non-vesicular mechanisms (by means of carrier proteins) and is targeted to the plasma membrane, thus maintaining a low ER cholesterol content. The trafficking between membranes through direct contact sites seems to be the easiest way to make cholesterol available to extracellular acceptors [11, 16].

**Deposition and cholesterol esters**

A surplus of cholesterol in neurons and other cell types is stored in the form of esters. Cholesterol esters constitute ~1% of the total cholesterol pool in the adult brain and exist as lipid droplets. A transient increase in esterified cholesterol concentrations, which accounts for over 5% of total cholesterol, is detected in a specific region of the brain at the onset of myelination. Cholesterol esters are a reserve pool of cholesterol and fatty acids which is utilized for the formation of myelin sheaths and synaptic contacts. The accumulation of cholesterol esters as cytoplasmic lipid droplets can result from increased Acyl-CoA cholesterol acyltransferase 1 gene expression (ACAT1, also named SOAT1), upregulated in response to high cholesterol levels in the ER. ACAT1 ablation leads to a decreased (by 86%) level of cholesterol esters. Conversely, neurotoxic agents and oxidative stress enhance ACAT1 activity [17]. ACAT1 is more abundantly expressed in neurons as compared to glial cells. Importantly, in astrocytes, ACAT1 is activated following impaired cholesterol efflux or in the presence of an excess of exogenous cholesterol [18]. Substrates for cholesterol esterification are provided by stearoyl-CoA desaturase, an ER enzyme that catalyzes the biosynthesis of monounsaturated fatty acids from saturated fatty acids [11].

Cytosolic cholesterol esters undergo degradation by hydrolyses. The concentration of cholesterol esters in the brain is maintained at a low level, and cholesterol hydrolase can convert the esters back to unesterified cholesterol. When cholesterol ester levels are dramatically raised, the enzyme fails to keep up with the cholesterol influx, allowing cholesterol ester droplets to build up in neuronal cytoplasm [1].

**Intercellular cholesterol trafficking**

## MATERIALS AND METHODS

The CNS cholesterol is transported to neurons via particles containing apolipoproteins (mainly ApoE, 39 kDa) and lipids. Astrocytes are the major source of cholesterol and apolipoprotein E (ApoE), which together with phospholipids forms lipoprotein complexes (ApoE-particles) (*Fig. 2*). The core of ApoE-particles is assembled in the ER, and the lipidation and secretion of ApoE are mediated by one or several ATP-binding cassette transporters (ABC), such as ABCA1, ABCG1, and ABCG4 [19-21]. ABCA1 catalyzes the transfer of cellular lipid to lipid-free apolipoproteins to generate nascent particles that undergo further lipidation, followed by ABCG1/ABCA1-mediated export from the cell [22]. Lipid-poor particles (for example, in the case of ABCA1 deficiency) show higher degradation rates, which leads to reduced ApoE levels in the brain. Mice in which ABCA1 has been specifically knocked out in the brain demonstrate cortical astrogliosis, increased inflammatory gene expression, as well as altered synaptic transmission and sensomotor behavior [23].

Lipoproteins are mainly targeted at neurons that are taken up by receptors belonging to the family of lowdensity lipoproteins (LDL): LDL-receptors and LDLreceptor- like proteins (LRP, LRP1B, LRP2/megalin, LRP4, LRP5/6, LRP8/APOER2, LRP11/SORL1). These receptors also bind the proteins involved in brain development (Sonic hedgehog, Wnt, reelin), including proteases, protease inhibitors (α2-macroglobulin), vitamin transporters, chaperones, and proinflammatory molecules [21]. LRP1 is an important receptor for ApoE-particles. It has a high transport capacity for ApoE due to the elevated rates of endocytic recycling (*Fig. 2*). LRP1 is primarily expressed in neurons; and the LDL-receptor – in glial cells [24]. Ablation of LRP1 function in neurons leads to global impairment of cholesterol homeostasis and neurodegeneration [25]. Following receptor-mediated endocytosis, vesicles deliver lipid particles to late endosomes/lysosomes. Immediately after endocytosis, ApoE is detached from lipid components and is not targeted at lysosomes but recycles back to the plasma membrane (*Fig. 2*) [26]. The liberated cholesterol exits late endosomes/lysosomes via NPC1-and NCP2-mediated pathways to reach the plasma membrane or the membrane of the ER, whereby the cholesterol content regulates the genes involved in cholesterol homeostasis via negative feedback (the SREBP-2/SCAP/INSIG-1 pathway) [16]. Within the endolysosome, cholesterol seems to be bound first by NPC2 (transmembrane protein), and then by NPC1 (intraluminal protein). In the bound state, cholesterol is shielded from the aqueous environment, followed by intracellular trafficking to the plasma membrane or the ER [27].

The interaction between ApoE-particles and receptors triggers multiple signaling networks essential to neuron survival and function [20, 21]. For example, ApoE upregulation in glial cells can accelerate nerve repair by 150-fold [28].

**Cholesterol excretion from the brain. Oxysterols**

Cholesterol is eliminated at a rate of 1g/day as bile acids (0.5 g) and cholesterol (0.5 g), as such, or coprostanol, a bacterially modified form. The brain lacks pathways for cholesterol degradation. However, cholesterol is exported from the brain at a rate of 6–12 mg/day (0.02–0.04% of total cholesterol turnover) [1] in the form of the brain-specific 24(S)-hydroxycholesterol (24-HC, 6–8 mg/day). The flux of 24-HC (in brain homogenate, 30 μM) out of the brain takes place by passing through the BBB (via diffusion or the anion transporting polypeptide 2, oatp2). Once in the circulation, it binds to LDL, is taken up by hepatocytes, and excreted in bile salts [7]. A small portion of cholesterol is removed from the brain as ApoE/A-particles via the BBB. Neuronal ABCA1 effluxes excess cholesterol to lipid-poor ApoA1, which are then transported through the BBB via LRP1 and scavenger receptor class B type 1 (SR1B) [29]. The upregulation or downregulation of neuronal ABCA1 expression can increase or reduce the cholesterol efflux, respectively [20].

The 24-HC production occurs by the action of cholesterol- 24-hydroxylase (CYP46A1), which is normally expressed in cell bodies and dendrites of nerve cells (large pyramidal cells of cortical areas, hippocampal cells, amygdale cells, putamen cells, thalamic cells, Purkinje cells) (*Fig. 2*) [7]. In disease or after trauma, CYP46A1 can be detected in non-nerve cells (astrocytes, microglia, macrophages) [11]. The brain-derived cholesterol metabolite 24-HC (as other oxysterols) stimulates the nuclear liver X receptor in astrocytes and neuron, inducing the expression of the proteins required for cholesterol biosynthesis and transport (ABCA1, ApoE). Following on from this, increased cholesterol excretion from the brain activates the mechanisms of *de novo *cholesterol synthesis and supply to neurons. When cholesterol levels in the ER membrane rise above a threshold, the expression of CYP46A1 is enhanced [1]. Overall, the brain engages in a rhythmic process of synthesis and export of cholesterol. However, using mice negative for CYP46A1 in the brain (CYP46A1 -/-mice, in which 24-HC levels were reduced by 95% when compared to 24-HC levels in wild-type mice), it was found that cholesterol levels remained fairly stable due to a 40–50% reduction in cholesterol production [7]. Overexpressing CYP46A1, which produces larger amounts of 24-HC, left cholesterol levels unaffected due to increased cholesterol synthesis [30]. The conversion of cholesterol into 24(S)-hydroxycholesterol in neurons is regulated by cholesterol etherification: therefore, ACAT1 gene ablation that reduces total brain cholesterol content by 13% increases 24-HC levels by 32% [17].

CYP46A1 can be activated by increased synaptic transmission. As early as 30 min after synaptic activity, the membrane cholesterol content in the glutamatergic synapse declines marginally but statistically significantly because of 24-HC release into the extracellular space. CYP46A1 moves from the ER to the plasma membrane and switches to the activated state. The activation depends on increases in cytosolic Ca^2+^ levels and STIM2, which detects changes in Ca^2+^ content stored in the ER [31]. Ageing is associated with increased production of reactive oxygen species that upregulate CYP46A1 expression, thus depleting cholesterol from synaptic membranes [32].

The other oxysterol is 27-HC, one of the major oxysterols in human circulation. Human physiological levels range typically from 0.15–0.73 μM, but under pathological conditions (e.g., atherosclerosis) they can reach millimole levels [33]. All body cells are involved in the synthesis of 27-HC, taking place in mitochondria via CYP27A1 (*Fig. 2*). Neurons, astrocytes and oligodendrocytes are capable of producing 27-HC, although to a very low extent. Clearance of 27-HC out of the brain occurs through the BBB [34]. However, 27-HC produced in peripheral tissues can gain access to the brain (5 mg/day). The normal ratio of 27-HC to 24- HC is 1 to 8 in the frontal cortex, 1 to 5 in the occipital cortex, and 1 to 10 in basal ganglia [35]. Oxysterol-7α- hydrolase (CYP7B1) catalyzes the conversion of 27- HC to 7α-hydroxy-3-oxo-4-cholestenoic acid, which is eliminated via the BBB [1]. High 27-HC levels have been affiliated with hypercholesteremia and oxidative stress [34]. Under oxidative stress conditions most brain cholesterol is metabolized into 27-HC, building up in the brain and increasing the risk of neurodegenerative diseases [33].

25-Hydroxycholesterol is a cholesterol metabolite that is produced and secreted by macrophages. The synthesis is catalyzed by cholesterol-25-hydrolase residing in the ER. In tissues (including nerve cells) the expression of the enzyme is upregulated in response to innate immunity stimuli. Recent studies have reported that 25-HC has an antiviral effect and promotes cholesterol esterification by increasing ACAT1 activity. The brain levels of 25-HC are approximately 1 μM and can be locally elevated in neurodegenerative disorders. It should be kept in mind that, tracing amounts of 25-HC can be synthesized by CYP46A1 and CYP27A1, and metabolized by CYP7B1 [36].

## CHOLESTEROL PATCHES IN THE BRAIN

**Brief characteristics of lipid rafts**

The plasma membrane organization of nerve cells has a more profound effect on cellular functionality than that of other cell types. Neurons and, to a lesser extent, glial cells are highly polarized cells having distinct membrane compartments: axon, dendrites, synaptic membranes, myelin sheaths, nodes, etc. Even within one membrane domain molecules are arranged in microdomains, termed lipid rafts, enriched in cholesterol and sphingolipids. Cholesterol functions as a dynamic “glue” that holds the microdomain assembly together [37]. Sphingolipids (in particular, glycolipids) are structurally variable, and certain neuron populations and glial cells can contain rafts composed of different glycolipids. During brain development and neural differentiation, a wide variety of glycolipids are increasingly expressed [3]. The impaired synthesis of complex glycolipids in neurons causes severe neural and synaptic defects, which ultimately ends up with death within 3 weeks of birth [38]. In general, the lipid composition of rafts in the brain varies with the area, cell type, and developmental stage. Certain rafts can contain protein components (receptors, ion channels, exo- and endocytic proteins, enzymes), which are recruited into lipid rafts to form signaling complexes/specialized subcompartments [3, 24]. High cholesterol content/raft density is one of the reasons behind the poor diffusion of proteins in synaptic membranes as compared to other cell types [39]. When cholesterol and sphingolipids levels are high in the membrane, lipid rafts coalesce into larger (micrometer-scale) and more stable raft clusters (platforms). Rafts are brought together by oligomerization of raft proteins by extracellular ligands (for example, growth factor) or cytoplasmic scaffolds. Phosphorylation increases the number of protein–protein interactions, which eventually influences the clustering. The merger of rafts is essential to membrane transport, signaling, and other cellular processes [3].

Rafts are associated with numerous proteins that have been implicated as regulators of signal transduction, including caveolins. The central segment of caveolin proteins contains the scaffolding domain that binds metabotropic receptors, G-proteins, NO-synthase, adenylate cyclase, phosphoinositol-3-kinase, MAP- and Src-kinases, protein kinase A and C [40]. In neurons, caveolin 1 is colocalized with the postsynaptic scaffolding protein PSD-95 and NMDA glutamate receptors. Caveolin- 1 knockout mice exhibit a loss of synapses [41]. Cerebral ischemia may disrupt the caveolin-associated signaling complexes present in neurons. Elevated caveolin- 1 expression leads to an increase in the activity of the signaling molecules that encourage the survival and growth of brain cells, conferring resistance to ischemic damage [40].

**Lipid rafts and intrinsically disordered proteins**

Proteins that lack a globular structure have been recognized and termed intrinsically disordered proteins. These proteins are highly abundant in eukaryotic proteomes, known as an unfoldome, and implicated in important cellular processes, such as signaling and membrane trafficking [42, 43]. The family of intrinsically disordered proteins includes α-synuclein, the amyloid precursor protein (APP), prion proteins (PrP), huntingtin protein (Htt), and tau protein. The structural organization of these proteins depends on the environment and is highly variable. Under certain conditions (overexpression, mutations, disfavored environment) α-synuclein, APP, and PrP are prone to acquiring a pathologic conformation. It is hypothesized that plasma membranes induce conformational changes of normal proteins into pathological forms. Once docked on the plasma membranes, the proteins are aggregated to form toxic oligomers. The amyloid-β peptide (proteolytically processed APP), α-synuclein, and PrP recognize specific components localized in lipid rafts, thereby further triggering their aberrant clustering [44]. The amyloid-β peptide can bind to glycosphingolipids (GM1 ganglioside, GM1 asialo-ganglioside, galactosyl ceramide) and cholesterol, α-synuclein to GM1 and GM3 gangliosides, PrP to sphingomyelin, galactosyl ceramide, GM1, and GM3 gangliosides [42]. These lipid raft components prevail in synaptic membranes [45]. The local pH value, cholesterol content, and membrane fluidity influence the strength of the interaction and aggregation state (globular or fibrillary), which ultimately determines the toxicity of the aggregate. Cholesterol increases or reduces the binding to sphingolipids containing non-hydroxylated/hydroxylated acyl groups. When GM1 ganglioside levels rise and the cholesterol and protein content drops in lipid rafts, the amyloid-β peptide assembles to form toxic fibrils on the plasma membrane, whereas an increase in membrane cholesterol concentrations inhibits the aggregation of the amyloid-β peptide [46]. Plasma membranes can mediate the transition of mature amyloid fibrils (with low toxic effects) into neurotoxic protofibrillar forms [47]. In other words, amyloid plaques, which contain inert plaque filaments, can be resolubilized into less longer structures – the soluble amyloid protofibrils. Oligomerized amyloid-β peptides contain a structural domain capable of binding to lipid raft-associated receptors such as NMDA glutamate receptors, mGluR5 Metabotropic Glutamate Receptor, and PrP. Oligomer binding triggers the clustering of specific lipid raft proteins into aberrant pathogenic platforms at the synapse [24, 44]. Furthermore, intrinsically disordered proteins themselves can exert deleterious effects on rafts and membranes, e.g., by depleting membrane cholesterol [42, 43].

## CHOLESTEROL AND SYNAPTIC TRANSMISSION

**Fig. 3:**
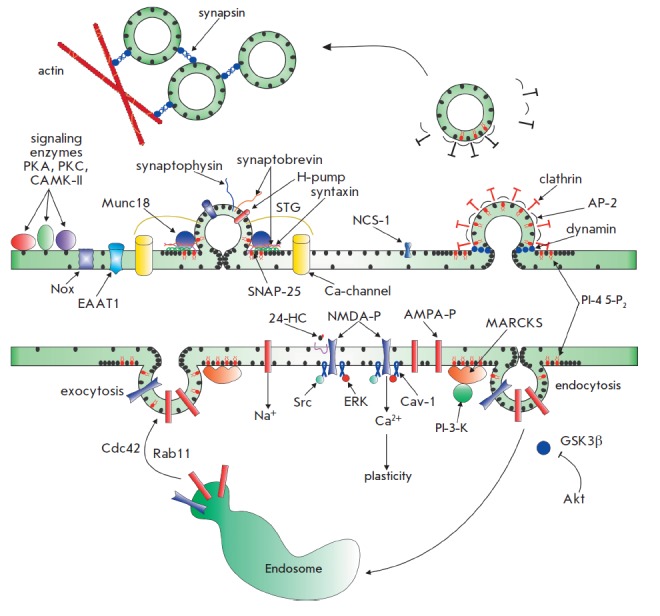
Synaptic transmission: lipid-protein interactions. The neurotransmitter is released from the synaptic vesicles upon fusion (exocytosis) with the presynaptic membrane at a specific site (termed active zone) in response to Ca^2+^ influx via potential-gated Ca-channels. The vesicle fusion is governed by proteins forming a SNARE complex (synaptobrevin, syntaxin, SNAP-25) and is dictated by numerous cholesterol-binding proteins (synaptotagmin, Munc-18, NCS-1) and signaling molecules (protein kinases, NADPH-oxidase/Nox). After fusion, the protein and lipid components of the vesicles undergo clathrin-mediated endocytosis. The vast majority of synaptic vesicles form the reserve pool, which maintains neurotransmission during prolonged synaptic activity. These vesicles are translocated into the active zone through an actin-and synapsin-dependent pathway. Glutamate released from the synaptic vesicles changes the Na^+^/Ca^2+^ conductivity of the postsynaptic membrane by activating AMPA/NMDA receptors. The surface expression of the postsynaptic receptors is dependent on the exo-and endocytotic trafficking of these receptors, which is regulated by small GTPase (Rab11) and protein kinase (Cdc42, GSK3β, phosphoinositol-3-kinase/PI-3-K). The receptor-dependent signaling is associated with many proteins (Src, ERC, Cav-1). As illustrated in *Fig.2*. Cholesterol molecules and its clusters are shown in black, phosphoinositol-4,5-biphosphates (PI-4,5-P_2_,) in red, and cholesterol/PI-4,5-P2-binding proteins. See text for a detailed explanation.

A schematic representation of signal transduction at the synapse is illustrated in *Fig. 3*. The presynaptic nerve terminals contain vesicles filled with neurotransmitters. In response to the action potential-driven Ca^2+^ influx, through potential-dependent Ca^2+^ channels, synaptic vesicles fuse with the presynaptic membrane (exocytosis), allowing the neurotransmitter to diffuse across the synaptic cleft. Following release onto the postsynaptic membrane, the neurotransmitter activates and alters the postsynaptic membrane potential. The synaptic transmission is one of the highly ordered cell processes. The efficiency of signal transduction lays the basis for integrative phenomena and can support the survival and function of neurons [37].

**Presynaptic mechanisms and cholesterol**

The role of cholesterol in presynaptic processes that regulate the release of a neurotransmitter is linked to the impact on membrane biophysics, the direct interaction with the proteins implicated in exo-and endocytosis, and the contribution to lipid raft formation.

Synaptic vesicle exocytosis induces membrane curvature stress, the extent of which is determined by the lipid composition of the membrane. Cholesterol, constituting 40% of total lipids in synaptic vesicles, serves as a scaffold to stabilize the curved membrane domains formed during vesicle fission and budding [48]. The apparently fast transbilayer diffusion of cholesterol (flipflop) contributes to the relaxation of the bending energy and formation of fusion pores. Cholesterol promotes membrane merging by interacting with vesicular (synaptophysin) and presynaptic (syntaxin-1) proteins [37, 49]. The exocytic sites and membrane vesicles contain cholesterol-enriched lipid rafts [45]. The other constituents of lipid rafts are key vesicle proteins, such as the proton pump, synaptophysins, synaptotagmins, synaptophysins, SV2 and presynaptic membrane exocytic proteins, such as syntaxin, SNAP-25, synaptobrevin, Munc18, and potential-dependent Ca-channels [50]. The efficiency of exocytosis depends on the association exocytic proteins with the lipid rafts. Syntaxin isoforms can cluster into isoform-specific patches in the plasma membranes, depending on the cholesterol content that define the functional properties of sites where exocytosis occurs [51]. It is likely that membrane fusion steps are driven by the merger/separation of certain rafts. For example, although potential-dependent Ca-channels and SNARE-proteins, on the one hand, and NCS-1 protein (neuronal calcium sensor 1) that increases Cachannel activity, on the other hand, localize to different microdomains, coalescence of these rafts favors exocytosis by clustering the proteins [52]. Ceramidase mutants, named ‘slug-a-bed’(slab), exhibit impaired cholesterol distribution in the presynaptic membrane with strong reduction (by 70% ) in vesicle fusion. Anion lipids and phosphatidylinositol 4,5-bisphosphates may accumulate in lipids, affecting exocytic proteins and membrane reshaping [49]. The oxysterol 5α-cholestan- 3-one disrupts the integrity of lipid rafts at the synapse, inhibits exocytosis, and decreases the number of vesicles available for neurotransmission [53]. In general, even mild cholesterol depletion reduces the evoked neurotransmitter release during both low- and highfrequency activity [54, 55]. Disorders in cholesterol metabolism negatively affect clustering of synaptic vesicles and the ion currents that contribute to the action potential [56, 57].

Membrane cholesterol specifically potentiates the evoked exocytosis, whereas, conversely, spontaneous release of synaptic vesicles is arrested by cholesterol [54, 55, 58]. It cannot be ruled out that cholesterol controls spontaneous synaptic vesicle release by preventing excess both the protein kinase activity (such as A, C, CAMK II) and the NADPH-oxidase-ROS-TRPV1- channel-Ca^2+^-calcineurin pathway activation [58-60]. For this reason, spontaneous exocytosis is sensitive to membrane cholesterol reduction, which via enhancement of spontaneous release ultimately depletes population of synaptic vesicles, induces neurotransmitter receptor desensitization, and downregulates local protein biosynthesis. In addition, changes to the membrane cholesterol content increase non-vesicular neurotransmitter release at periphery and central synapses [61, 62].

*Endocytosis of synaptic vesicles. *Endocytosis of synaptic vesicles prevents synaptic vesicle pool depletion. Following endocytosis, vesicles are refilled with the neurotransmitter and recycle. Cholesterol regulates membrane invagination during endocytosis [37, 49]. Cholesterol-rich membrane domains contribute to the activation of endocytic proteins [50]. It is thought that the lipid rafts in the vesicular membranes prevent the mixing of vesicle proteins with presynaptic proteins to maintain proper sorting [45]. Raft phosphoinositides are implicated in the triggering of endocytic events and clustering of vesicular coat proteins [49]. Even slight cholesterol extraction from vesicle membranes blocks endocytosis and traps vesicular proteins in the plasma membrane [54, 62].

**Postsynaptic events and cholesterol**

Changes to the number and content of postsynaptic receptors are critical to synaptic plasticity, occurring through both lateral diffusion (between postsynaptic and extrasynaptic sites) and endo-exocytic traffic of these receptors (*Fig. 3*). Receptor trafficking is regulated by interaction with scaffold proteins and membrane lipid constituents [3]. Receptor activity and downstream signal transduction also depend on the membrane cholesterol content. Most postsynaptic receptors colocalize with lipid rafts [2, 3, 11, 12]. The postsynaptic density is a macromolecular assembly composed of the proteins responsible for postsynaptic signaling and membrane plasticity, attached to the lipid rafts [39, 63]. In this review, we will focus on AMPA- and NMDA-glutamate receptors.

Acute cholesterol depletion inhibits both currents through AMPA-receptors and AMPA receptor exocytosis [64]. Chronic depletion of cholesterol or sphingolipids upregulates constitutive AMPA receptor internalization [63]. Conversely, there is evidence that cholesterol reduction by 25% in naturally aging neurons causes accumulation of AMPA receptors on the cell surface, due to impaired endocytosis and lateral diffusion. In this scenario, cholesterol loss seems to provoke the detachment of MARCKS from membrane phosphoinositol- 4,5-biphosphates, which are then catalyzed by phosphoinositol-3-kinase into phosphoinositol-3,4,5- triphosphates that stabilize F-actin and increase Akt kinase activity. F-actin restricts post-synaptic AMPAreceptor mobility, and Akt kinase suppresses the glycogen synthase kinase 3β (GSK3β) implicated in AMPAreceptor endocytotic trafficking [12].

The oligomerization of NMDA-receptors is favored by their localization in rafts. Cholesterol depletion inhibits Ca^2+^ influxes via NMDA-receptors, promotes their desensitization, and suppresses long-term potentiation in the hippocampus [65]. Conversely, 24-HC allosterically potentiates an NMDA receptor-mediated response, facilitating long-term potentiation in hippocampal slices. Interestingly, 25-HC (in a submicromolar range) counteracts the effect of 24-HC [66]. The influx of Ca^2+^ through NMDA-receptors can drive both synaptic plasticity and cell death (excitatory toxicity), depending on the amplitude of Ca^2+^ influx and receptor colocalization (within or outside rafts, in synaptic or extrasynaptic space). NMDA-receptors associated with rafts interact with caveolin-1, which is required for inducing the Src-kinase/ERK-kinase pathway to promote survival in neurons. Hence, their contribution to excitory toxicity is minimized. Upon long-term exposure to agonist and ischemia, NMDA-receptors are trafficked to the non-raft membrane [67]. Overactivation of extrasynaptic NMDA-receptors has a profound impact on excitatory toxicity [12]. Lipid rafts contain the excitatory amino acid transporter EAAT1-4, and cholesterol loss reduces Na+-transporter-mediated glutamate uptake by glial cells and neurons [68] to trigger excitatory toxicity. Importantly, activated NMDA-receptors rapidly deplete the intracellular pool of cholesterol (probably, recycling endosomes), thereby activating Cdc42- and Rab11-dependent trafficking of AMPA-receptors to the postsynaptic membrane. This participates in the induction of long-term synaptic potentiation [69].

## CHOLESTEROL AND NEURODEGENERATIVE DISORDERS

A growing body of evidence supports a role for cholesterol biosynthesis defects and impaired synaptic transduction in neurodegenerative disorders [2, 11, 12]. The importance of cholesterol in the brain has been revealed in many of the less common neurological disorders secondary to mutations in genes for cholesterol biosynthesis enzymes. We performed a literature search to collect evidence on alterations of cholesterol metabolism in CNS disorders associated with mutations in the genes implicated in the biosynthesis of cholesterol (Smith-Lemli-Opitz syndrome), intracellular trafficking (Niemann–Pick type C disorder), and synthesis regulation (Huntington’s disease).

**Smith-Lemli-Opitz syndrome**

**Fig. 4:**
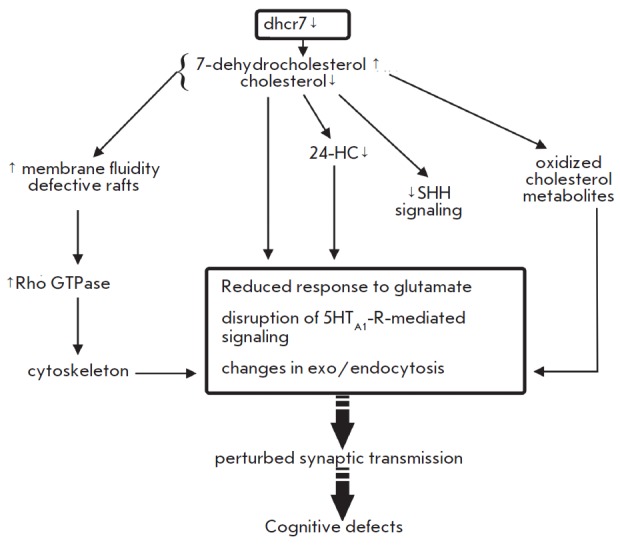
Alterations in cholesterol synthesis associated with the Smith- Lemli-Opitz syndrome: an implication in synaptic dysfunction. See text for a detailed explanation.

For some diseases, cholesterol metabolism abnormalities are the major cause of neurodegenerative disorders and birth defects. Lathosterolosis is a retardation syndrome due to a deficiency in 3β-hydroxysteroid-5- desaturase, and desmosterolosis is caused by mutations in the 3β-hydroxysterol-24-reductase gene. Defective cholesterol-27-hydroxylase leads to cerebrotendinous xanthomatosis. The Smith-Lemli-Opitz syndrome is the most common autosomal recessive disease of this type (affects 1 in 20,000 newborns) resulting from mutations in the dhcr7 gene encoding 7-dehydrocholesterolreductase (Dhcr7) [70]. Severe forms are deleterious for fetal development and newborn infant. Dhcr7 catalyzes the final step in the Kandutsch–Russell cholesterol biosynthetic pathway. A consequence of defective Dhcr7 is the accumulation of 7-dehydrocholesterol (7DHC) in the brain, non-neuronal tissues and plasma, and ultimately, cholesterol loss (*Fig. 4*). In the Smith- Lemli-Opitz syndrome, 24-HC levels drop and 27-HC levels increase in the plasma [71]. This disease involves profound brain development abnormalities, intellectual disability, as well as emotional and sleep disorders. Patients with severe cases display plasma cholesterol concentrations amounting to 2% of the normal range. In the mild form, plasma cholesterol levels may remain unaffected but that cannot stop brain developmental defects, pointing to a role for brain cholesterol in the genesis of neurological symptoms [70]. On the other hand, these symptoms could result from the accumulation of the Dhcr7 substrate 7,8-dehydrodesmosterol and its oxidized derivatives [72]. The teratogenic activity in the Smith-Lemli-Opitz syndrome seems to be attributed to a deficiency in SHH-signaling, since SHH activity (Sonic Hedgehog morphogen) requires a covalent linkage of cholesterol to SHH [70].

In the Smith-Lemli-Opitz syndrome, while cholesterol shares physicochemical properties with 7-DHC, cholesterol is depleted from the membrane and replenished with 7-DHC. These subtle changes in membrane composition lead to reduced membrane stiffness and lower the ability to stabilize the membrane curvature that affect the fussion/fission. 7-DHC can also produce defective membrane rafts with abnormal protein interface [73]. A reduced cholesterol availability has a negative effect on the signaling functions mediated via multiple receptors. Mutant mice with the Smith- Lemli-Opitz syndrome exhibit an impaired response of NMDA-receptors to glutamate [74]. 7-DHC is sensitive to oxygen, generating 7-DHC-derived oxysterols, which are active in the low micromolar range [72]. As a consequence, endo- and exocytosis could be influenced [55]. Ligand binding by serotonin(1A) receptors can be suppressed by 7-DHC [75]. In the Smith-Lemli-Opitz syndrome, hippocampal axons and dendrites exhibit increased activation of Rho GTPases (involved in actin cytoskeleton dynamics) due to the altered raft compositions [76]. Another hallmark of the Smith-Lemli-Opitz syndrome is increased phosphorylation of cofilin-1, which fails to act as an actin depolymerizing factor. Cytoskeleton stabilization could indirectly affect endo- and exocytosis, as well as trafficking of synaptic vesicles and receptors. Taken together, these alterations disrupt the release of a neurotransmitter (serotonin, dopamine) and ultimately lead to a neurological disease [77].

**Niemann–Picktype C disorder**

**Fig. 5:**
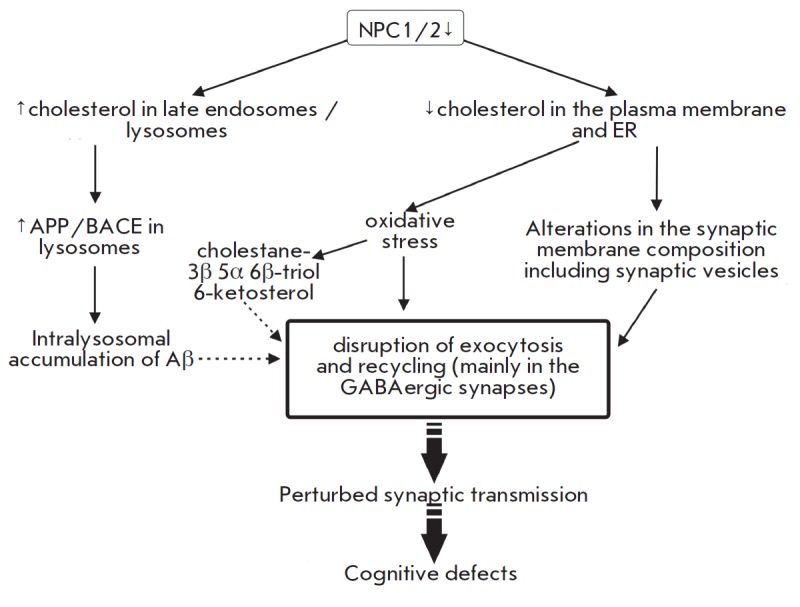
Changes in cholesterol metabolism in Niemann Pick disease type C: the impact on synaptic transmission. See text for a detailed explanation.

There is clear evidence linking a disordered cholesterol metabolism to brain neurodegeneration. Niemann– Pick type C is a rare autosomal recessive disorder (affecting 1 in 150,000 newborns) characterized by progressive neuronal death and reduced life expectancy, hepatolienomegaly, and lung deficiency. The Niemann–Pick type C disease is accompanied by early loss of Purkinje cells in the cerebellum, leading to ataxia [2]. Mutations in either the NPC1 (95% of cases) or the NPC2 (5% of cases) gene render the encoded proteins non-functional (*Fig. 5*). Defective NPC1 or NPC2 in neurons and glial cells do not allow cholesterol and other lipids (glycolipids, in particular) to exit late endosomes/ lysosomes and traffic to the plasma membrane and the ER [16]. In NPC1-deficient neurons, cholesterol is dramatically decreased in distal axons but accumulates in soma of neurons. It is likely that the defects seen in the Niemann–Pick type C disorder are caused by a reduced cholesterol content in axons: presynaptic nerve terminals, in particular. This is consistent with the evidence indicating an altered composition and organization of synaptic vesicles and recycling endosomes in nerve terminals with dysfunctional NPC1 [18]. The Niemann–Pick type C disorder is accompanied by an elevated accumulation of oxysterols, such as 3β,5α,6β- cholestantriol and 6-ketosterol, in the brain as a result of oxidative stress [2].

Neuronal soma degeneration is the final event in the pathological cascade of the Niemann–Pick type C disorder. At the onset of neurodegeneration, presynaptic nerve terminals undergo degeneration, followed by accumulation of defective NPC1 in recycling endosomes [18]. Neurodegeneration seems to originate from nerve terminals. At early stages of the disease (prior to neurological abnormalities and synapse losses) presynaptic impairments occur in the form of suppression of the evoked exocytosis and insufficient delivery of synaptic vesicles to exocytic sites [78]. These events are more severe in GABAergic nerve terminals, resulting in an imbalance of inhibitory and excitatory processes [79]. It is tempting to speculate that the alterations affecting the synaptic transmision trigger the abnormalities observed in the Niemann–Pick type C disorder manifested by ataxia, cataplexy, and a loss of reflexes. Similar changes in exocytosis of synaptic vesicles occur upon extraction of membrane cholesterol with concentrated methyl-β-cyclodextrin (MβCD) [78]. Detection of NPC1 in recycling endosomes of nerve terminals suggests a role for NPC1 in the slow vesicle recycling critical to the maintenance of synaptic vesicles during prolonged synaptic activity [18].

There is no therapeutic option currently available for the treatment of the Niemann–Pick type C disorder. However, recent work raises hope for a possible intervention. Notably, a single subcutaneous administration of the cholesterol-binding agent MβCD to animals with deleted NPC1 activity delayed the development of neurodegeneration and doubled the lifespan [80]. Although MβCD cannot cross the BBB, very small amounts do pass into the brain. High levels of MβCD (5–10 mM), commonly utilized for cholesterol depletion, are toxic to neurons and block synaptic transduction [3]. Low doses of MβCD (0.1 mM) barely effect membrane cholesterol and could be taken up in neurons [62]. Endocytosed MβCD seems capable of extracting the sequestered cholesterol from the endosome/ lysosome compartment and translocating it to the ER and the plasma membrane. In another study, it was discovered that injecting 2-hydroxypropyl-β-cyclodextrin into the cerebrospinal fluid reduces cholesterol accumulation in endo/lysosomes and rescues Purkinje cells [16]. Polyrotoxanes, a new class of compounds, have been shown to undergo lysosomal degradation to form β-cyclodextrines that are capable of preventing lysosomal sequestration of cholesterol [81]. It should be kept in mind that they also show neuroprotective activity in cell and mouse models of Alzheimer’s disease [82].

Neurons with Niemann-Pick Type C demonstrate β amyloid peptide accumulation (in cholesterol-laden lysosomes) and fibrillar globules of an abnormally hyperphosphorylated tau protein. The cerebrospinal fluid carries high levels of amyloid β 38, 40, and 42 species. Notably, affected individuals lack amyloid plaques, because the disorder is fatal within the first years of life [83]. Diffuse amyloid deposits occur in subjects with the ApoE4 allele, which disturbs amyloid β clearance. The ApoE4 allele carrier status is associated with increased disease severity and early onset of neurological symptoms [84].

**Huntington’s disease**

**Fig. 6:**
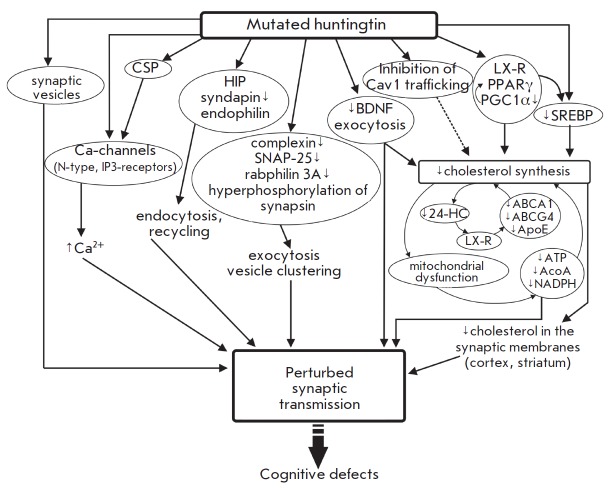
Influence of the mutant huntingtin on synaptic transduction and brain cholesterol metabolism. See text for a detailed explanation.

Huntington’s disease is an autosomal dominant neurodegenerative disease accompanied by cognitive and motor dysfunction. Huntington’s disease is caused by a genetic defect leading to an expansion of a polyglutamine stretch (over 36 residues, polyglutamine expansion) in the target protein, named huntingtin. Striatum and cortex neurons are sensitive to the toxic property of the mutated protein [85]. Huntington’s disease is associated with reduced cholesterol synthesis in the brain [10]. The mutant protein huntingtin decreases the transcriptional activity of SREBP, downregualting SREBPregulated genes and, in turn, the cholesterol biosynthetic pathway in cortical and striatum neurons (*Fig. 6*). Cholesterol levels are first affected in synaptosomal membranes and, at later stages, in myelin sheaths. Exogenous cholesterol (up to 15 μM) prevents the death of striatal neurons carrying the mutant protein [86]. There is a strong positive correlation between longer polyglutamine stretches and the severity of diseases and cholesterol biosynthesis disorder [34]. Huntington’s disease rodents show a dramatic age-related decline in cholesterol content in brain tissue as compared to agematched healthy individuals [86]. Cholesterol levels in fibroblasts are reduced by 50%, along with lowered total plasma cholesterol concentrations, which are detectable early in pre-manifest patients [87]. Conversely, 24-HC levels are first elevated at the onset of diseases and later dropping due to dysfunction of cholesterol biosynthesis in degenerating striatal neurons [10]. The first increase in 24-HC content could represent a response to compensate for cholesterol loss. A further decline in 24-HC levels in the brain leads to reduced cholesterol synthesis because of downregulation of LX-receptors and, correspondingly, LX-receptor-dependent protein expression (ABCA1, ABCG4, ApoE). Astrocytes bearing the mutant huntingtin produce and secrete less ApoE. Such ApoE-particles are smaller in size and lipid-poor, failing both to efficiently transfer cholesterol from astrocytes to neurons and to clear cholesterol excess from the brain [86]. LX-receptor agonists can partially ameliorate Huntington’s disease symptoms [10]. In cholesterol-deficient cells, cholesterol and its esters can form patches in plasma membranes and lysosomes/endosomes, due to prevention in efflux in the form of ApoE-particles and 24-HC. Aberrant cholesterol accumulation could be a result of defects in caveolin 1 traffic induced by the mutant huntingtin[ 88]. BDNF released by nerve terminals of cortical neurons in the striatum is implicated in not only synaptic plasticity and cell survival, but also induction of cholesterol synthesis in postsynaptic neurons. The mutant huntingtin inhibits cholesterol synthesis by influencing trafficking and secretion of BDNF [10].

The intact huntingtin can bind to the nuclear receptors engaged in lipid metabolism such as LX-receptor, PPARγ (peroxisome-proliferator-activated receptor gamma), and vitamin D receptor [10]. Overexpressed wild-type huntingtin activates LX-receptors, whereas in the absence of the huntingtin, LX-receptor-mediated transcription issuppressed . It is likely that mutant huntingtin plays a little or no role in triggering LXreceptor- dependent networks, including SREBP. In oligodendrocytes, mutant huntingtin impairs the ability of PPARγ CoActivator 1α (PGC1α) to enhance both the cholesterol biosynthetic pathways andexpression of myelin proteins [89]. In the pre-manifest stage of the disease, PGC1α expression is lowered in medium spiny neurons of the striatum. This may be one of the reasons for mitochondrial dysfunction, since PGC1α underlies mitochondrial biogenesis and oxidative metabolism and modulates the expression of the genes encoding proteins of the electron transport chain [90]. Mitochondrial dysfunction causes ATP and NADPH depletion, reducing availability of important substrate for cholesterol synthesis. The mutant protein significantly affects the membrane fluidity probably via cholesterol loss. Olesoxime, a cholesterol-like compound capable of passing into the cells and then trapped in the mitochondrial membrane, has been recently shown to exert a therapeutic effect in the correction of mitochondrial dysfunction in disease models of amyotrophic lateral sclerosis, peripheral neuropathies, and Huntington’s disease. HD cells exposed to olesoxime showed a decrease in membrane fluidity, whereas long-term exposure elevates the cholesterol content [91].

In the pre-manifest stage, exo-endocytic trafficking of synaptic vesicles is perturbed (Fig. 6). The huntingtin protein builds up at the presynapse and binds to synaptic vesicles. Huntington’s disease mice show abnormal synapsin-1 phosphorylation and progressive decline in the concentration of complexin II, SNAP-25, and rabphilin 3A in nerve terminals of select areas of the cortex [92]. As a consequence, exocytosis is suppressed and the pool of synaptic vesicles is reduced. The level of intracellular Ca^2+^ in terminals is elevated due to the attenuation of tonic Ca^2+^ channel inhibition by CSP (cysteine-string protein), modulating N-type Ca^2+^ current, or directly by huntingtin, regulating IP3-receptors of ER [93]. There is a group of endocytic proteins tightly binding to huntingtin, including HIP1 (huntingtin interacting protein 1), HIP1R, syndapin I, and endophilin. Syndapin I, an endocytic scaffolding protein, is eliminated from presynaptic regions, and HIP1 becomes dysfunctional and blocks endocytosis. In addition, Huntington’s disease is accompanied by defective Rab11-dependent endosomal recycling, causing abnormalities in the generation of synaptic vesicles and synaptic transmission [94].

## Abbreviations

ABC: ATP-binding cassette transporters
ACAT1: acetyl coenzyme A cholesterol acyltransferase
ApoE: apolipoprotein E
BDNF: brain derived neurotrophic factor
HMGCoA: hydroxy-3-methylglutaryl-coenzyme A
HC: hydroxycholesterol
BBB: blood-brain barrier
Dhcr7: 7-dehydrocholesterol reductase
LDL-receptor: low-density lipoprotein receptor
LRP: LDL-receptor related protein
LX-receptor: Liver X receptor
MCD: methyl-β-cyclodextrin
CYP46A1: cholesterol 24-hydroxylase
CYP27A1: cholesterol 27-hydroxylase
CYP7B1: oxysterol 7α-hydrolase
ER: endoplasmic reticulum
